# Depletion of CpG Dinucleotides in Papillomaviruses and Polyomaviruses: A Role for Divergent Evolutionary Pressures

**DOI:** 10.1371/journal.pone.0142368

**Published:** 2015-11-06

**Authors:** Mohita Upadhyay, Perumal Vivekanandan

**Affiliations:** Kusuma School of Biological Sciences, Indian Institute of Technology Delhi, New Delhi, 006, India; Albert Einstein College of Medicine, UNITED STATES

## Abstract

**Background:**

Papillomaviruses and polyomaviruses are small ds-DNA viruses infecting a wide-range of vertebrate hosts. Evidence supporting co-evolution of the virus with the host does not fully explain the evolutionary path of papillomaviruses and polyomaviruses. Studies analyzing CpG dinucleotide frequencies in virus genomes have provided interesting insights on virus evolution. CpG dinucleotide depletion has not been extensively studied among papillomaviruses and polyomaviruses. We sought to analyze the relative abundance of dinucleotides and the relative roles of evolutionary pressures in papillomaviruses and polyomaviruses.

**Methods:**

We studied 127 full-length sequences from papillomaviruses and 56 full-length sequences from polyomaviruses. We analyzed the relative abundance of dinucleotides, effective codon number (ENC), differences in synonymous codon usage. We examined the association, if any, between the extent of CpG dinucleotide depletion and the evolutionary lineage of the infected host. We also investigated the contribution of mutational pressure and translational selection to the evolution of papillomaviruses and polyomaviruses.

**Results:**

All papillomaviruses and polyomaviruses are CpG depleted. Interestingly, the evolutionary lineage of the infected host determines the extent of CpG depletion among papillomaviruses and polyomaviruses. CpG dinucleotide depletion was more pronounced among papillomaviruses and polyomaviruses infecting human and other mammals as compared to those infecting birds. Our findings demonstrate that CpG depletion among papillomaviruses is linked to mutational pressure; while CpG depletion among polyomaviruses is linked to translational selection. We also present evidence that suggests methylation of CpG dinucleotides may explain, at least in part, the depletion of CpG dinucleotides among papillomaviruses but not polyomaviruses.

**Conclusions:**

The extent of CpG depletion among papillomaviruses and polyomaviruses is linked to the evolutionary lineage of the infected host. Our results highlight the existence of divergent evolutionary pressures leading to CpG dinucleotide depletion among small ds-DNA viruses infecting vertebrate hosts.

## Introduction

Small ds-DNA viruses have a genome size of less than 10 kb and are dependent on host cell machinery for their replication. Papillomaviruses and polyomaviruses represent small ds-DNA viruses infecting vertebrates. Papillomaviruses have circular, ds-DNA genomes of approximately 8 kb in length. Papillomaviruses are diverse in nature and are known to infect mammals including humans, birds and reptiles. These viruses cause benign and malignant tumors. The life cycle of papillomavirus depends on the host cell it infects. During replication, papillomaviruses may integrate into the host genome.

Cross-species infections and recombination are uncommon among papillomaviruses [1]. Evidence for co-evolution with the host does not fully explain the evolutionary path of papillomaviruses [1]. The evolutionary rates (nucleotide substitutions per site per year) reported for papillomaviruses vary over orders of magnitude (10^−7^ to 10^−9^) [2, 3, 4]; nonetheless, all reports in literature suggest slow evolutionary rates for papillomaviruses. Major synonymous codon usage bias is known to exist among papillomaviruses [5]. The link between codon usage bias and gene expression has been well documented among papillomaviruses [6]. However, it remains unclear whether the codon usage bias is driven by translational selection or mutational pressure.

Polyomaviruses have a circular ds-DNA genome of approximately 5 kb in length. Like papillomaviruses, polyomaviruses also infect a wide-range of vertebrate hosts including humans, mammals and birds. According to the International committee on taxonomy of viruses (ICTV), polyomavirus can be divided into three genera: (a) Orthopolyomavirus (b) Wukipolyomavirus and (c) Avipolyomavirus [7]. Mammalian polyomaviruses usually infect only a specific host species [7]. In contrast, avian polyomaviruses have the ability to infect multiple hosts and are able to replicate in a variety of tissues or organs [8].

Features shared between papillomaviruses and polyomaviruses include (a) temporal gene regulation [9, 10] (b) the presence of overlapping reading frames [11, 12] (c) presence of both integrated and extra-chromosomal forms of the virus [13, 14] and (d) ability to cause cancer. In addition, evidence for and against co-evolution with the infected hosts [15, 16] are reported for both the groups of viruses.

Understanding the evolution of papillomaviruses and polyomaviruses remains an area of intense research. Most studies have focused on sequence comparison, phylogenetics and substitution rates. These studies provide interesting insights on evolutionary rates, co-evolution with the host, evolutionary timelines and also predict the origin and spread of viruses; nonetheless, they do not provide clues on the underlying evolutionary pressures.

A limited number of studies have analyzed dinucleotides in virus genomes. Studies on the relative abundance of dinucleotides in virus genomes provide novel perspectives on evolutionary pressures driving virus evolution [17, 18, 19]. The CpG dinucleotide is the most extensively studied dinucleotide in virus genomes. Depletion of CpG dinucleotides has been reported among several DNA and RNA viruses [19, 20]. Methylation of cytosines within the CpG dinucleotide followed by deamination leading to a C to T substitution [19] resulting in the loss of CpG dinucleotides to minimize the stimulation of toll like receptor 9 is believed to be the cause of CpG dinucleotide depletion among DNA viruses [21]. In contrast, the factors leading to CpG depletion among RNA viruses remain unclear. Small DNA viruses infecting humans including papillomaviruses and polyomaviruses are reported to be CpG depleted [17]. However, CpG depletion has not been extensively investigated among papillomaviruses and polyomaviruses.

The number of full-length sequences available for polyomaviruses and papillomaviruses has increased exponentially in the last 10 years. In addition, a sizable number of new human and animal polyomaviruses have been discovered recently. In this study, we investigate the relative abundance of dinucleotides among papillomaviruses and polyomaviruses. We also aim to analyze differences, if any in the extent of CpG dinucleotide depletion among different vertebrate host lineages. In addition, we attempt to identify the predominant evolutionary pressures leading to CpG depletion among papillomaviruses and polyomaviruses. We believe that our findings will provide new insights on the evolutionary pressures shaping papillomaviruses and polyomaviruses.

## Materials and Methods

### Retrieval of sequences

All full-length sequences of viruses belonging to the family *Papillomaviridae* and *Polyomaviridae* available in the NCBI viral genome resources (http://www.ncbi.nlm.nih.gov/genome/viruses/) were retrieved for analysis. When multiple full-length sequences were available for a particular virus, only one full-length virus sequence was used for analysis. A total of 183 sequences were used for the analysis; this includes 127 full-length sequences from the family *Papillomaviridae* and 56 full-length sequences from the family *Polyomaviridae*. The accession numbers of the viruses studied are provided in S1 Table.

### Calculation of dinucleotide frequencies

The observed/expected frequency for the dinucleotide (XpY) in single-stranded DNA organisms is calculated using the formula: (O/E)_XpY_= [*f*(XY)/f(X) f(Y)]* G [19]

Where *f*(XY) is the frequency of the dinucleotide XpY, *f*(X) and *f*(Y) are the frequencies of mononucleotides X and Y respectively and *G* is the genome length.

For organisms with double-stranded sequences, the complementary nucleotide strand should also be considered for calculating the observed/expected frequencies for dinucleotides. In other words, in a double-stranded sequence, frequency of dinucleotide XpY in one strand will be equal to the frequency of dinucleotide Y’pX’ in the complementary strand, where Y’ and X’ are complementary nucleotides to Y and X respectively.

Thus, the dinucleotide frequencies in a double-stranded sequence is calculated using the formula:

$$\left(\frac{O}{E}\right)XpY=\left(\frac{O}{E}\right)Y^{\prime }pX^{\prime }=\frac{2\left(fXpY+fY^{\prime }pX\prime \right)}{\left(fX+fY\right)\left(fX^{\prime }+\;fY^{\prime }\right)}$$

where, XpY denotes the dinucleotide in one strand, Y`pX`denotes the complementary dinucleotide in the opposite strand; *f*(X), *f*(Y), *f*(X`) and *f*(Y`) are the frequencies of mononucleotides X, Y, X`and Y`respectively; *f*(XpY) and *f*(Y`pX`) are the dinucleotide frequencies of XpY and Y`pX`[22].

### Calculation of codon usage frequencies

Effective number of codon (ENC), total GC content and the nucleotide composition at the third codon position was determined using a web tool Codon W (http://mobyle.pasteur.fr/cgi-bin/portal.py#forms::CodonW). ENC values range from 20 (where only one codon is used for one amino acid) representing maximum codon usage bias to 61 (where all the codons are equally used for each amino acid) representing no codon usage bias. The expected ENC value (ENC*) was calculated by using the following formula: ENC*= 2+GC_3_ + {29/[(GC_3s_)^2^ + (1−GC_3s_)^2^]} [23]. The influence of GC content on codon usage bias was determined using the ENC-GC_3_ plot [23].

Relative synonymous codon usage (RSCU) is used to determine the number of times a codon appears in a gene divided by the expected frequency under equal codon usage. If the synonymous codons of an amino acid are used with equal frequencies, the RSCU value will be 1. When the RSCU value is greater than 1, the codons have positive codon usage bias and if the value of RSCU is less than 1, the codons have negative codon usage bias.

The relationship between GC content at the third codon position (GC_3_) and GC content at the non-synonymous codon positions (GC_1,2_) was studied to determine the influence of translational selection and mutational pressure on virus evolution.

### Calculation of dinucleotide frequencies in the coding regions

The coding DNA sequences (CDS) and intergenic regions as annotated in Genbank files were extracted using a web tool (http://www.cbs.dtu.dk/services/FeatureExtract/). The distribution of dinucleotide (XpY) in CDS and non-coding regions (intergenic and terminal regions) of each genome was calculated.

### Statistical analysis

Data were analyzed using Mann Whitney U test, Wilcoxon signed rank test, Pearson’s correlation coefficient (*r* ^*2*^) as appropriate. All the graphs were made using MS-Excel or the software Graph pad. Scatter plots were used to compare two parameters. Results were considered statistically significant at a *P* value of <0.05.

## Results and Discussion

### Evolutionary lineage of the infected host is linked to the extent of CpG depletion

**Fig 1:**
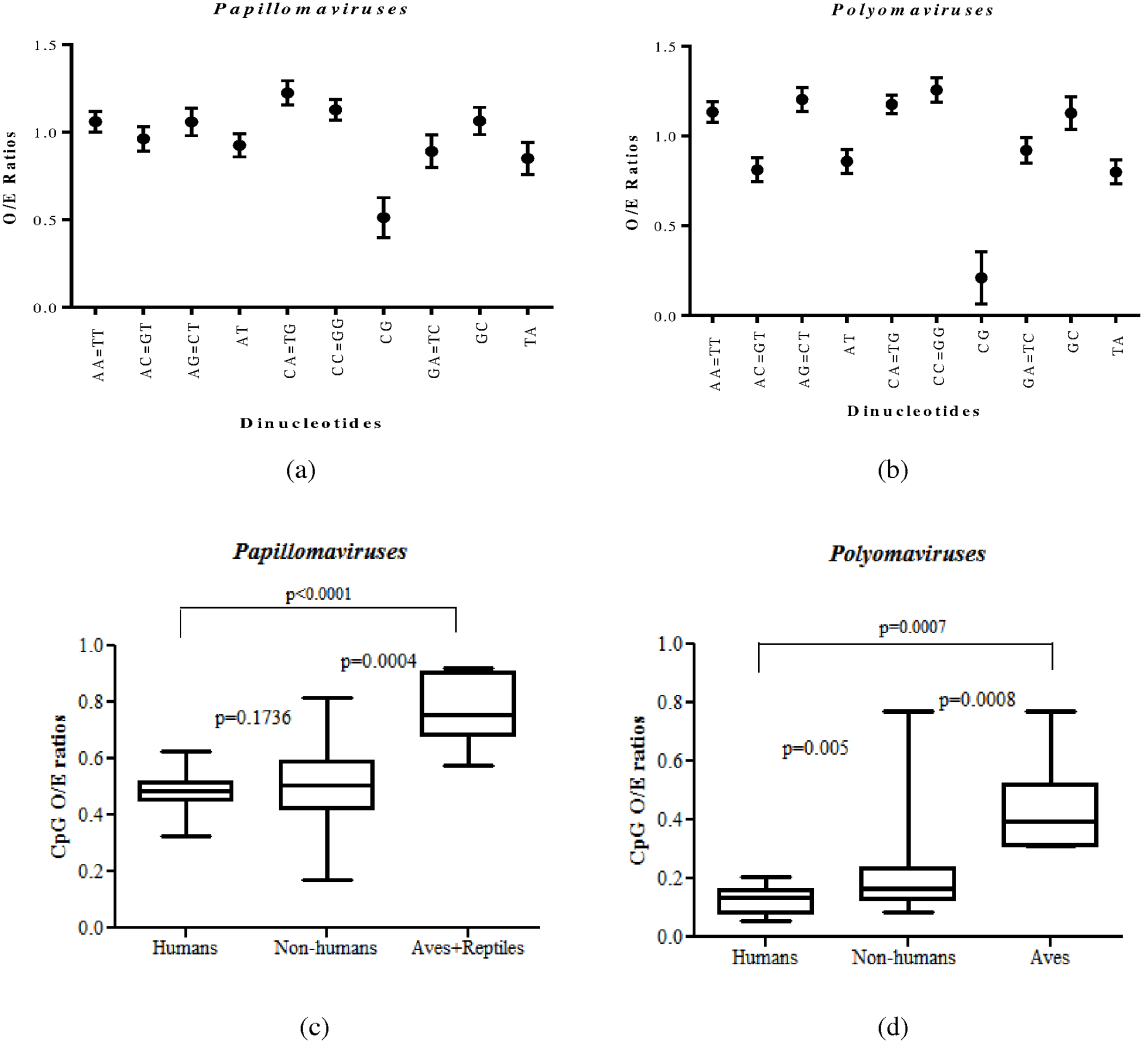
Relative abundance of dinucleotides in papillomaviruses and polyomaviruses. (a) The mean value for each dinucleotides O/E ratio (closed circles) are plotted for papillomaviruses. Among all the dinucleotide, CpG dinucleotides were clearly depleted among papillomaviruses (b) The mean value for each dinucleotides O/E ratio (closed circles) are plotted for polyomaviruses. CpG dinucleotide depletion is pronounced among polyomaviruses. (c) Among papillomaviruses, those infecting humans or other mammals (mammals other than humans) had significantly lower CpG O/E ratios than those infecting aves/reptiles [0.48 (95% CI of 0.47 to 0.5) vs 0.77(95% CI of 0.67 to 0.87); P<0.0001 and 0.51 (95% CI of 0.48 to 0.54) vs 0.77(95% CI of 0.67 to 0.87) P = 0.0004]. The relative abundance of CpG dinucleotides among papillomaviruses infecting humans was marginally lower than that of those infecting other mammals, this difference was not significant [0.48 (95% CI of 0.47 to 0.5) vs 0.51 (95% CI of 0.48 to 0.54), P = 0.1736]. (d) Polyomaviruses infecting humans were significantly CpG depleted as compared to those infecting other mammals [0.12(95% CI of 0.1 to 0.15) vs 0.20(95% CI of 0.16 to 0.24); P = 0.005] or aves [0.12(95% CI of 0.1 to 0.15) vs 0.44(95% CI of 0.3 to 0.57); P = 0.0007].

Dinucleotide frequencies among the viruses that belong to the family *Papillomaviridae* and *Polyomaviridae* are summarized in Fig 1a and 1b respectively. Since our study pertains to ds-DNA viruses, there are a total of 10 unique dinucleotides instead of 16 dinucleotides; for example the relative abundance of the dinucleotide GpT in one strand will be the same as the relative abundance of ApC in the complementary strand [18]. The average O/E ratios for dinucleotides in viruses belonging to the family *Papillomaviridae* and the family *Polyomaviridae* are shown in Fig 1a and 1b respectively. Clearly, CpG dinucleotide was the most depleted dinucleotide as compared to any other dinucleotide among papillomaviruses and polyomaviruses (P<0.0001; Fig 1a and P<0.0001; Fig 1b). TpA dinucleotides were the second most depleted dinucleotides (CpG being the most depleted dinucleotide) in both the groups of viruses (P<0.0001; Fig 1a and 1b). TpA dinucleotides are generally depleted among viruses [19, 20]. The presence of UpA in two stop codons [24] and the increased susceptibility of UpA to ribonuclease digestion [25] are believed to lead to TpA depletion. The CpG O/E ratios observed for polyomaviruses were significantly lower than those observed for papillomaviruses [0.21(95% CI of 0.17–0.25) vs 0.51(95% CI of 0.49–0.53); P<0.0001; Fig 1a and 1b].

The distribution of CpG O/E ratios for papillomaviruses and polyomaviruses infecting different host groups is shown in box plots in Fig 1c and 1d respectively. Among papillomaviruses, those infecting humans or other mammals (mammals other than humans) had significantly lower CpG O/E ratios than those infecting aves/reptiles [0.48(95% CI of 0.47 to 0.5) vs 0.77(95% CI of 0.67 to 0.87); P<0.0001 and 0.51(95% CI of 0.48 to 0.54) vs 0.77(95% CI of 0.67 to 0.87); P = 0.0004; Fig 1c]. While the relative abundance of CpG dinucleotides among papillomaviruses infecting humans was marginally lower than that of papillomaviruses infecting other mammals, this difference was not significant [0.48 (95% CI of 0.47 to 0.5) vs 0.51 (95% CI of 0.48 to 0.54); P = 0.17; Fig 1c].

Among polyomaviruses, the lowest CpG O/E ratio was observed among those infecting humans [0.12 (95% CI of 0.1 to 0.15); Fig 1d]. Polyomaviruses infecting humans were significantly CpG depleted as compared to those infecting other mammals [0.12 (95% CI of 0.1 to 0.15) vs 0.20(95% CI of 0.16 to 0.24); P = 0.005; Fig 1d] or aves [0.12 (95% CI of 0.1 to 0.15) vs 0.44 (95% CI of 0.3 to 0.57); P = 0.0007; Fig 1d].

CpG depletion has been reported among few human and non-human papillomaviruses and polyomaviruses [17]. Our data suggests that in both the groups of viruses studied severe CpG depletion is seen among those infecting humans or other mammals; while modest CpG depletion is seen among those infecting aves or reptiles. Here, we demonstrate for the first time that the extent of CpG depletion among papillomaviruses and polyomaviruses is dependent on the evolutionary lineage of the infected host; thus implying a role for host-induced pressures in the depletion of CpG dinucleotides. Understanding and correcting for phylogenetic inertia may help better understand the link between CpG dinucleotide depletion and the evolutionary lineage of the infected host. Nonetheless, there is no consensus on the methods to measure phylogenetic inertia, precluding meaningful corrections for phylogenetic inertia in the data analysed. While our results suggest host evolutionary lineage-dependent depletion of CpG dinucleotides in both the groups of viruses studied, it is not possible to rule out if the observed differences in CpG dinucleotides are linked to inheritance of CpG depleted DNA; this may be particularly relevant for non-mammalian papillomaviruses that are monophyletic.

### Depletion of CpG dinucleotides: a potential role in the evolution of papillomaviruses and polyomaviruses

**Fig 2:**
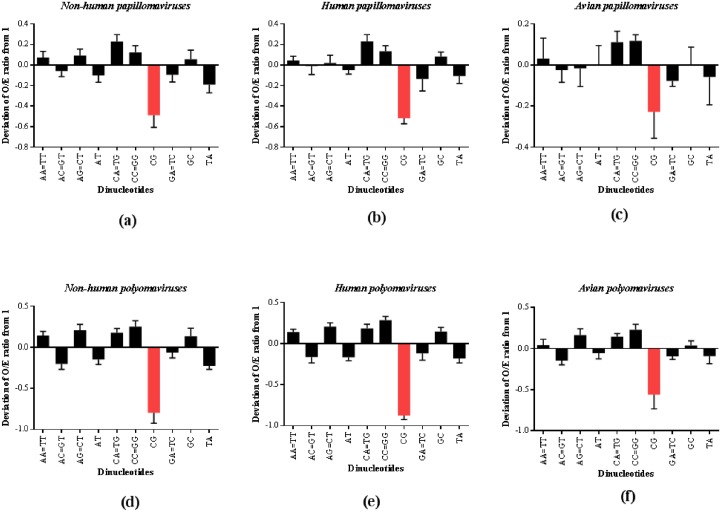
Role for depletion of CpG dinucleotides in the evolution of papillomaviruses and polyomaviruses. CpG dinucleotides are the most deviant (the difference between expected frequency and observed frequency) dinucleotides among papillomaviruses infecting (a) mammals (non-humans) (b) humans and (c) aves/reptiles. Similarly, CpG dinucleotides are the most deviant dinucleotides among polyomaviruses infecting (d) mammals (non-humans), (e) humans and (f) aves. This finding supports a major role for CpG dinucleotide depletion in the evolution of papillomaviruses and polyomaviruses across different host groups. Red color represents the deviation of CpG dinucleotides from 1 across all host groups of papillomaviruses and polyomaviruses.

CpG dinucleotide depletion appears to be most pronounced among papillomaviruses and polyomaviruses infecting humans and mammals, while those infecting aves/reptiles appear to be less amenable to CpG depletion. We then analyzed the deviation of the dinucleotide O/E ratios from 1 (the O/E ratio will be 1 if the observed number of dinucleotides is equal to the expected number of dinucleotides) among the different host groups infected by papillomaviruses and polyomaviruses. This analysis clearly demonstrates that CpG dinucleotides are the most deviant (the difference between expected frequency and observed frequency) dinucleotides across all host groups of papillomaviruses and polyomaviruses (Fig 2). Despite modest depletion of CpG dinucleotides among papillomaviruses infecting aves/ reptiles and polyomaviruses infecting aves, the loss of CpG dinucleotides predominates over the loss or gain of any other dinucleotide. This finding suggests a major role for CpG dinucleotide depletion in the evolution of all papillomaviruses and polyomaviruses including those infecting aves or reptiles.

### Evolutionary lineage of the infected host and synonymous codon usage bias of CpG-containing codons

**Fig 3:**
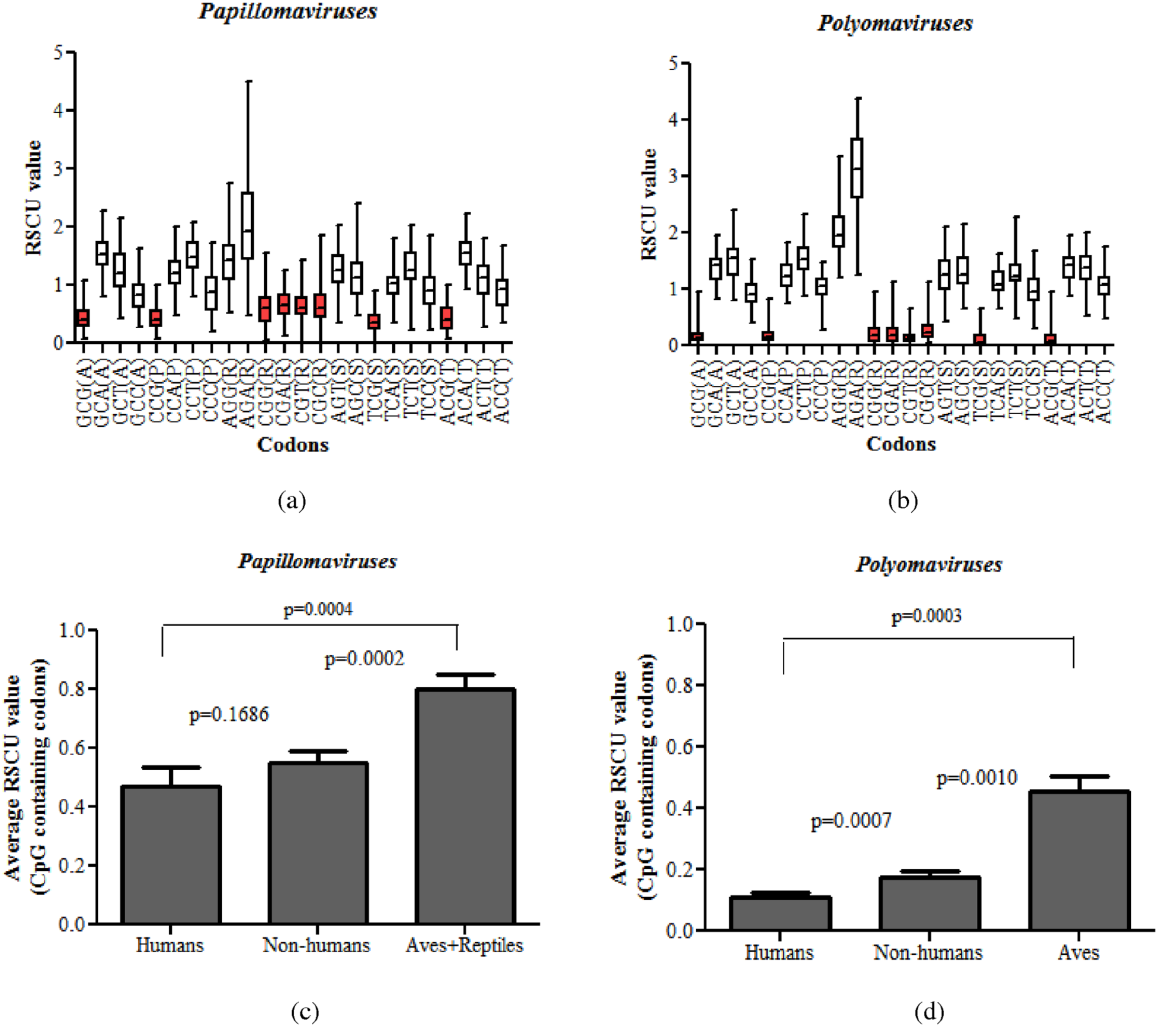
Relative synonymous codon usage (RSCU) values of CpG-containing codons. Box plots showing the RSCU values of CpG-containing codons among (a) papillomaviruses and (b) polyomaviruses. CpG-containing codons are shown in red colour. The encoded amino acid is shown in parenthesis. Both group of viruses avoided CpG-containing synonymous codons as 100% (8 out of 8) of these codons had an RSCU value below one. (c) RSCU values of CpG-containing codons among papillomaviruses infecting different host groups: papillomaviruses infecting humans or other mammals had significantly lower RSCU values than those infecting aves/reptiles [0.47(95% CI of 0.42 to 0.52) vs 0.8(95% CI of 0.68 to 0.92); P = 0.0004; 0.55(95% CI of 0.53 to 0.58) vs 0.8(95% CI of 0.68 to 0.92); P = 0.0002]. (d) RSCU values of CpG-containing codons among polyomaviruses infecting different host groups: polyomaviruses infecting humans had significantly lower RSCU values as compared to those infecting other mammals [0.11(95% CI of 0.09 to 0.14) vs 0.17(95% CI of 0.16 to 0.19), P = 0.0007] or aves [0.11(95% CI of 0.09 to 0.14) vs 0.45(95% CI of 0.34 to 0.57); P = 0.0003].

The extent of CpG dinucleotide depletion varies greatly across papillomaviruses and polyomaviruses. Nonetheless, all papillomaviruses and polyomaviruses are CpG depleted. In order to analyze CpG-containing synonymous codon usage preferences we studied the RSCU values of CpG-containing synonymous codons. Amino acids for which none of the synonymous codons contained CpG dinucleotides were not analyzed. Clearly, both group of viruses avoided CpG-containing synonymous codons as 100% (8 out of 8) of CpG-containing synonymous codon had an RSCU value below one (Fig 3a and 3b).

The distribution of RSCU values for CpG-containing codons in both groups of viruses infecting different host groups is shown in Fig 3c and 3d. Papillomaviruses infecting humans or other mammals had significantly lower RSCU values than those infecting aves/reptiles [0.47(95% CI of 0.42 to 0.52) vs 0.8(95% CI of 0.68 to 0.92); P = 0.0004; 0.55(95% CI of 0.53 to 0.58) vs 0.8 (95% CI of 0.68 to 0.92); P = 0.0002; Fig 3c]. Polyomaviruses infecting humans had significantly lower RSCU values as compared to those infecting other mammals [0.11(95% CI of 0.09 to 0.14) vs 0.17(95% CI of 0.16 to 0.19), P = 0.0007; Fig 3d] or aves [0.11(95% CI of 0.09 to 0.14) vs 0.45(95% CI of 0.34 to 0.57); P = 0.0003; Fig 3d].

The RSCU values for CpG-containing synonymous codons in papillomaviruses and polyomaviruses infecting different host groups (Fig 3c and 3d) are in keeping with the extent of CpG dinucleotide depletion (Fig 1c and 1d). This finding suggests that synonymous codon usage may reflect the relative abundance of dinucleotides. Synonymous codon usage bias may be influenced by genome-wide mutational pressure [26] or translational selection in coding DNA sequences [27]. Synonymous codon usage bias of CpG-containing codons among papillomaviruses and polyomaviruses infecting different host groups may be linked to the evolutionary lineage of the infected host. To the best of our knowledge, host evolutionary lineage-related differences in codon usage bias have not been reported among viruses infecting vertebrates.

Preference for thymine at the third codon position has been reported among papillomaviruses in synonymous codons encoding 14 amino acids [28]; however, the underlying mechanism is not well understood. Synonymous codon usage preferences among polyomaviruses have not been studied. Our findings suggest that CpG-containing synonymous codons are avoided by both papillomaviruses and polyomaviruses

### A role for mutational pressure in the evolution of papillomaviruses and polyomaviruses

**Fig 4:**
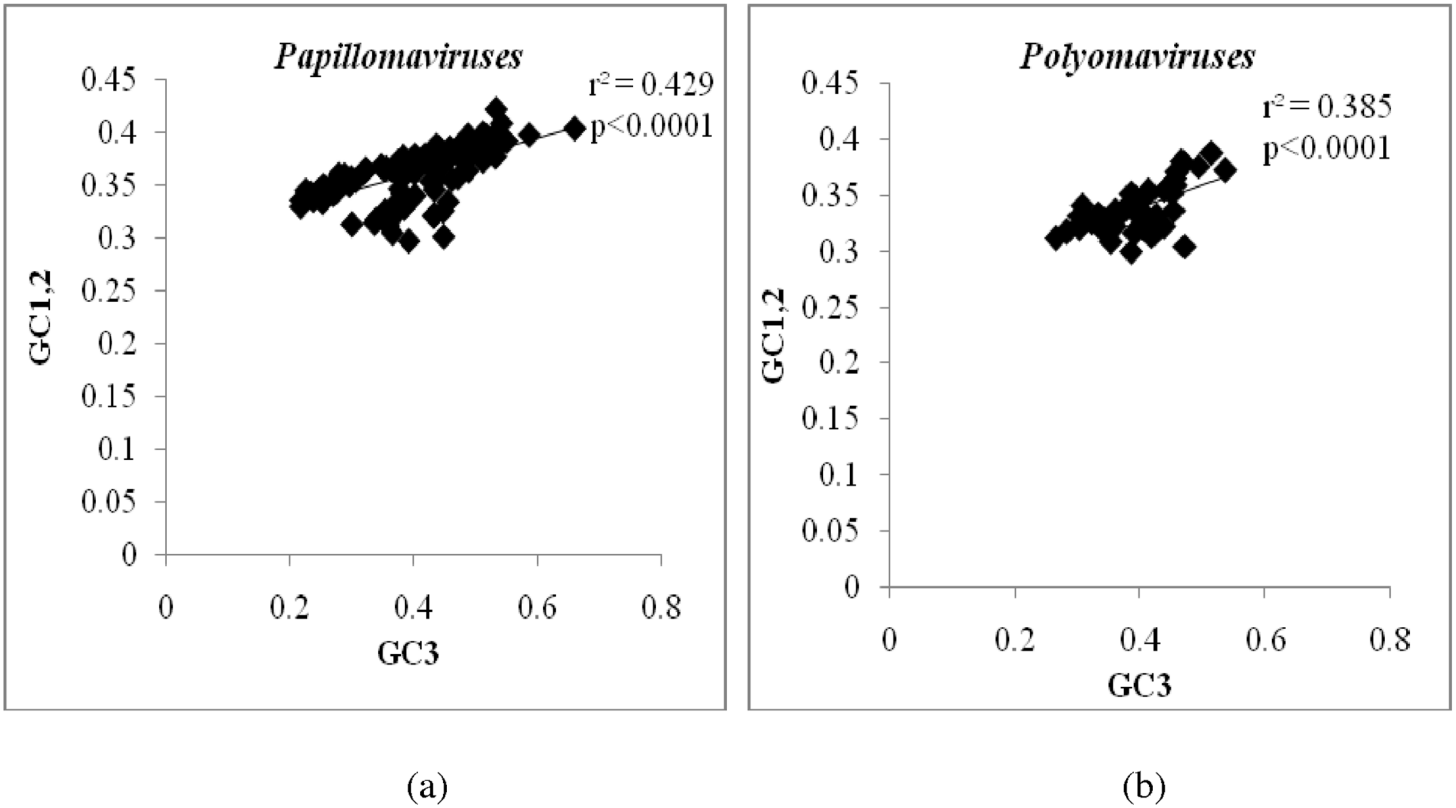
Role for mutational pressure in the evolution of papillomaviruses and polyomaviruses. Scatter plot demonstrating a good correlation between GC content at the third codon position (GC_3_) (X-axis) and GC content at first and second codon position (GC_1,2_) (Y-axis) among (a) papillomaviruses and (b) polyomaviruses. This finding suggests that mutational pressure contributes to the evolution of both papillomaviruses and polyomaviruses.

To investigate the role of mutational pressure in the evolution of papillomaviruses and polyomaviruses, we first analysed the correlation between GC content at first and second codon positions (GC_1,2_) and GC content at third codon position (GC_3_). Mutational pressure, if present will not act on specific codon positions and will therefore similarly affect GC_1,2_ and GC_3_. A good correlation between GC_1,2_ and GC_3_ implies a role for mutational pressure in virus evolution. We found a significant correlation between GC_1,2_ and GC_3_ among papillomaviruses (R^2^ = 0.429; P<0.0001; Fig 4a) and polyomaviruses (R^2^ = 0.385; P<0.0001; Fig 4b), suggesting that mutational pressure contributes to the evolution of both papillomaviruses and polyomaviruses.

### Translational selection is pronounced among polyomaviruses

**Fig 5:**
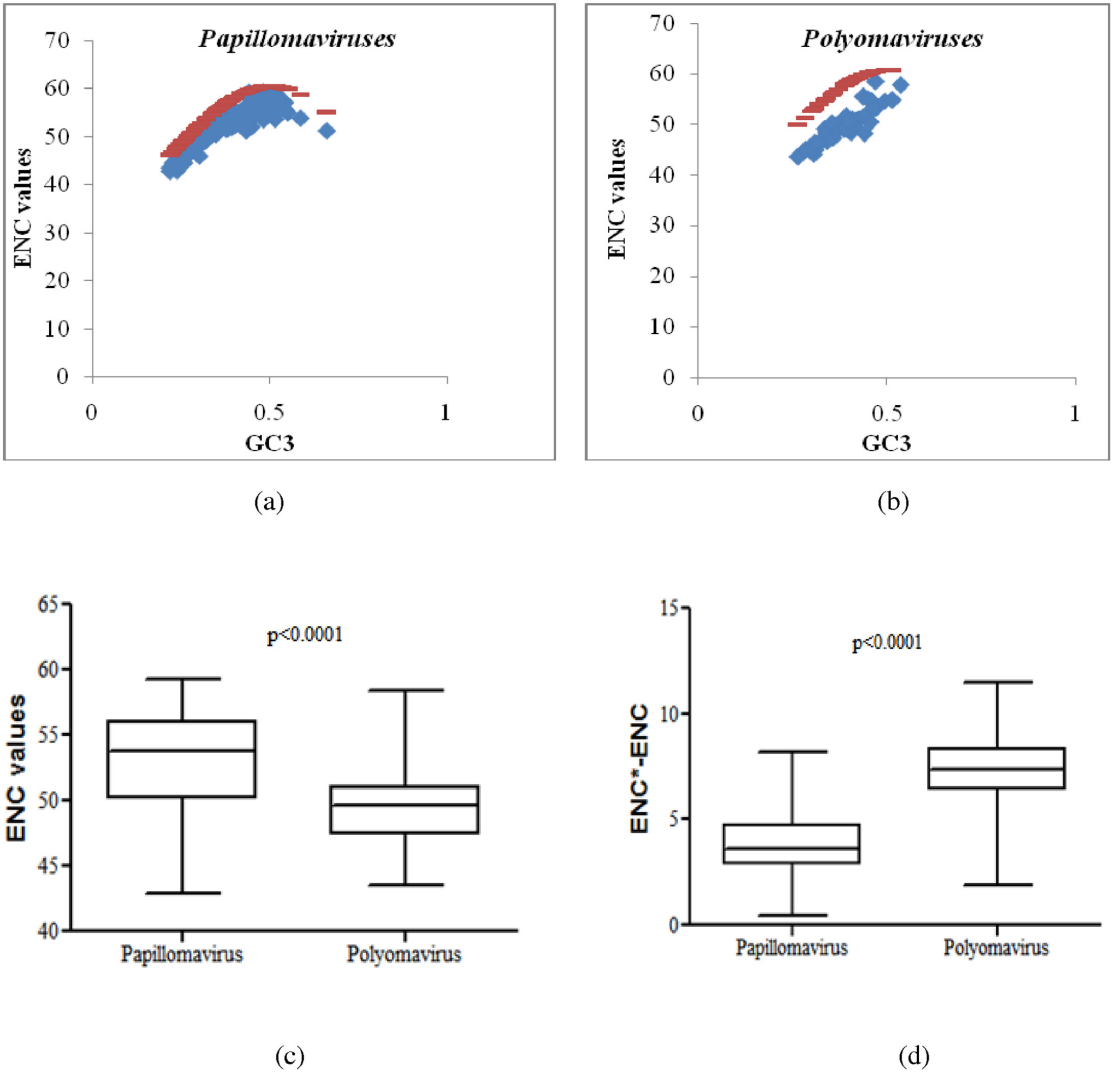
Translational selection is more pronounced among polyomaviruses. Correlation between ENC values and GC_3_ among (a) papillomaviruses (b) polyomaviruses. The red line represents the ENC expected value (ENC*) and blue diamonds represent the ENC values. The ENC values for papillomaviruses lie on, or just below the ENC expected curve whereas, the ENC values for polyomaviruses lie well below the ENC expected curve. (c) Box plots comparing the ENC values of papillomaviruses and polyomaviruses. Codon usage bias is more pronounced among polyomaviruses as compared to papillomaviruses as indicated by lower ENC values among polyomaviruses [49.74(95% CI of 48.87 to 50.61) vs 52.79 (95% CI of 52.06 to 53.54); P<0.0001]. (d) Box plots showing the differences between the expected ENC values (ENC*) and actual ENC values. The differences expected ENC values (ENC*) and actual ENC values were significantly higher among polyomaviruses as compared to papillomaviruses [7.30(95% CI of 6.86 to 7.75) vs 3.78 (95% CI of 3.54 to 4.03); P<0.0001]; this finding confirms increased codon usage bias or translational selection among polyomaviruses.

We then studied the role of translational selection in the evolution of papillomaviruses and polyomaviruses. We first used effective number of codon (ENC) statistic for analysing the codon usage bias. ENC values range from 20–61 and lower the ENC value higher the codon usage bias [18]. The ENC values ranged from 42.87–59.23 [mean: 52.79(95% CI range 52.06 to 53.54)] for papillomaviruses and from 43.51–58.38 [mean: 49.74(95% CI of 48.87 to 50.61)] for polyomaviruses. We also analysed the relationship between ENC and GC_3_ (ENC-GC_3_ plot). Three parameters are plotted on an ENC-GC_3_ plot: ENC values, expected ENC values and the GC_3_ content within codons. ENC values are on or just below the ENC expected values when synonymous codon usage bias is absent or minimal; in contrast, if ENC values are well below the ENC expected curve it indicates the presence of major synonymous codon usage bias or translational selection. In our study, we found that the ENC values for papillomaviruses lie on, or just below the ENC expected curve (Fig 5a) whereas, the ENC values for polyomaviruses lie well below the ENC expected curve (Fig 5b).

The ENC values clearly indicate a stronger codon usage bias among polyomaviruses as compared to papillomaviruses [49.74(95% CI of 48.87 to 50.61) vs 52.79(95% CI of 52.06 to 53.54); P<0.0001; Fig 5c]. This finding suggests that translational selection is more pronounced among polyomaviruses as compared to papillomaviruses. The GC content of the genome is known to influence ENC values [29], however the formula for calculating the expected ENC values corrects for the differences in GC content [23]. Papillomaviruses have significantly higher GC content as compared to polyomaviruses (P = 0.0047; S1 Fig). It is therefore possible that the observed differences in ENC values between the two groups of viruses could potentially be influenced by the differences in the GC content of their genomes. We therefore analysed the difference between the expected ENC value and the actual ENC value. The differences between expected ENC values and actual ENC values were significantly higher among polyomaviruses as compared to papillomaviruses [7.30(95% CI of 6.86 to 7.75) vs 3.78 (95% CI of 3.54 to 4.03); P<0.0001; Fig 5d]; this finding confirms that the increased codon usage bias / translational selection among polyomaviruses as compared to papillomaviruses is independent of differences in GC content.

Single amino acid changes have been reported to alter tissue tropism [30], pathogenesis [31] and the phenotype [32] among polyomaviruses suggesting a potential role for translational selection as an evolutionary force in shaping polyomaviruses.

### CpG depletion: A role for divergent evolutionary pressures

Our findings clearly demonstrate that CpG dinucleotides are the most depleted dinucleotides across papillomaviruses and polyomaviruses. To understand the relative roles of mutational pressure and translational selection as driving forces leading to the loss of CpG dinucleotides we investigated the differences in the CpG dinucleotide O/E ratios between coding DNA sequences (CDS) and non-coding DNA sequences. The difference in dinucleotide O/E ratio between the coding and the non-coding regions for a given dinucleotide may help assess the predominant evolutionary force leading to the loss or gain of the dinucleotide. For example, if translational selection is the predominant evolutionary force leading to the loss of CpG dinucleotides, CpG dinucleotides will be more depleted in the coding DNA sequences as compared to the non-coding region of the genome and hence the CpG O/E ratios for the CDS will be lower than that for the non-coding region.

The differences between coding and the non-coding dinucleotide O/E ratios for CpG dinucleotides among papillomaviruses and polyomaviruses are shown in Table 1. Among papillomaviruses the relative abundance of CpG dinucleotides was comparable between the CDS and the non-coding DNA sequences [0.51 (95% CI of 0.49 to 0.53) vs 0.52 (95% CI of 0.48 to 0.56); P = 0.8131; Table 1], suggesting that mutational pressure is the predominant driving force leading to the depletion of CpG dinucleotides among this group of viruses. In contrast, among polyomaviruses the CpG dinucleotide O/E ratios for the CDS were significantly lower than that for the non-coding regions [0.19 (95% CI of 0.15 to 0.23) vs 0.26 (95% CI of 0.22 to 0.31); P = 0.0001; Table 1], vindicating that CpG depletion in this group of viruses is primarily driven by translational selection.

**Table 1:** CpG dinucleotide frequencies in coding DNA sequences and non-coding sequences.

	CpG O/E ratio in non-coding region	CpG O/E ratio in CDS	Wilcoxon signed rank test	Inference
Papillomaviruses	0.52(95% CI: 0.48 to 0.56)	0.51(95% CI: 0.49 to 0.53)	P = 0.8131	Mutational pressure
Polyomaviruses	0.26(95% CI: 0.22 to 0.31)	0.19(95% CI: 0.15 to 0.23)	P = 0.0001	Translational selection

Interestingly, different evolutionary pressures lead to the CpG dinucleotide depletion among the small ds-DNA viruses infecting vertebrates. We have reported a role for DNA methylation-linked mutational pressure as the mechanism driving CpG depletion among parvoviruses [19]. Studies investigating the evolutionary forces driving CpG depletion are limited. Polyomaviruses are the amongst the most CpG depleted viruses reported in literature; however, the evolutionary forces underlying CpG depletion among polyomaviruses have not been investigated. Here we report translational selection as the primary evolutionary force driving CpG depletion among polyomaviruses.

### Methylation of CpG dinucleotides may partially explain CpG dinucleotide depletion among papillomaviruses

**Fig 6:**
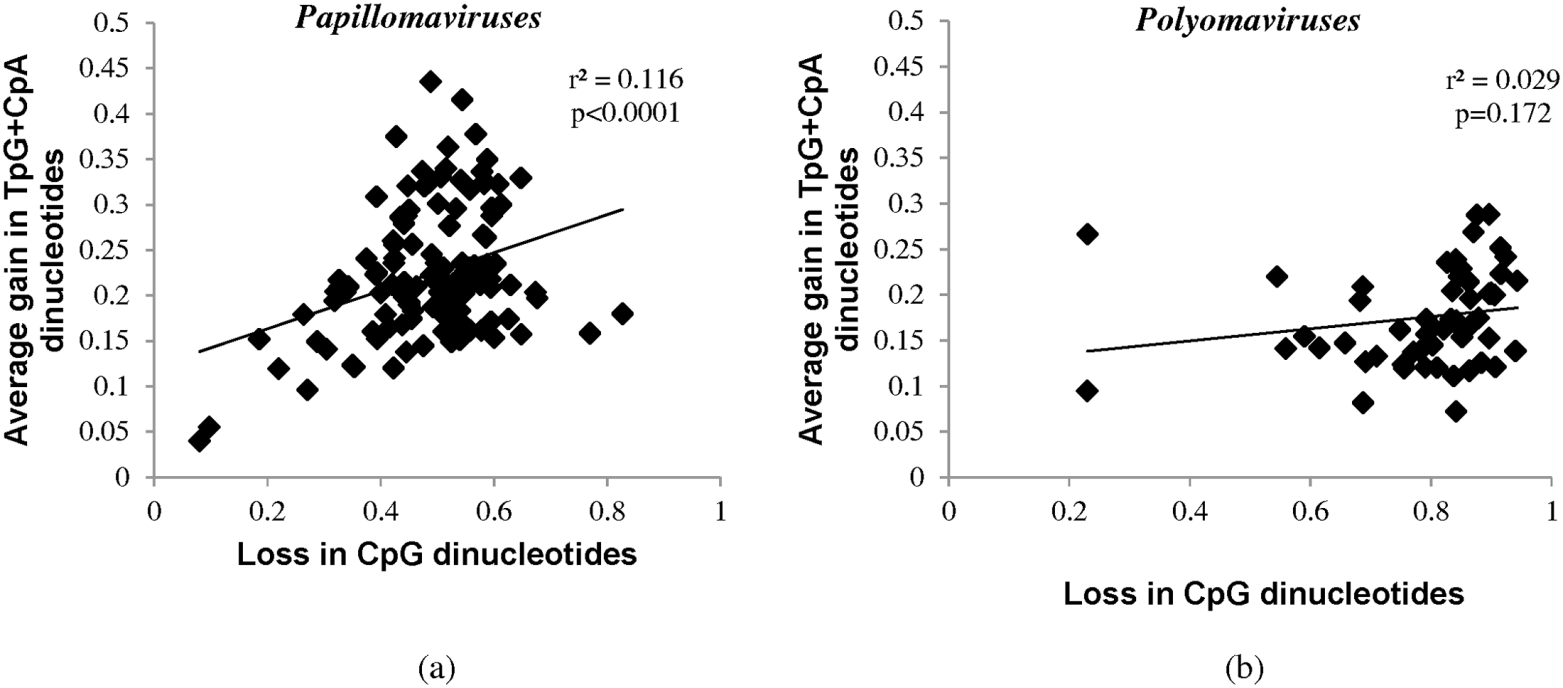
Methylation of CpG dinucleotides may partially explain CpG dinucleotide depletion among papillomaviruses. (a) Scatter plot demonstrating a weak but significant correlation between the loss of CpG dinucleotides (X-axis) and the average gain in TpG and CpA dinucleotides (Y-axis) among papillomaviruses (R² = 0.116; P<0.0001). (b) Scatter plot demonstrating the lack of correlation between the loss of CpG dinucleotides (X-axis) and the average gain in TpG and CpA dinucleotides (Y-axis) (R² = 0.029; P = 0.172).

Deamination of 5-methylcytosine (5mC) leads to C to T transition [33], resulting in the depletion of CpG dinucleotides. The depletion of CpG dinucleotides by deamination of 5mC within the CpG dinucleotide results in a gain of TpG (in the same strand) and CpA (in the complementary strand) dinucleotides [34]. A correlation between the loss of CpG dinucleotides and the gain in TpG and CpA dinucleotides has been used as a surrogate to assess CpG depletion by methylation and subsequent deamination [19]. Interestingly, we found a significant, albeit weak, correlation between the loss of CpG dinucleotides and the average gain of TpG and CpA dinucleotides among papillomaviruses (R² = 0.116; P<0.0001; Fig 6a). In contrast, among polyomaviruses there was no correlation between the loss of CpG dinucleotides and the average gain in TpG and CpA dinucleotides (R² = 0.029; P = 0.172; Fig 6b). Our findings suggest that methylation of cytosines within CpG dinucleotides followed by deamination of methylated cytosines to thymines accounts at least in part for CpG depletion among papillomaviruses but not among polyomaviruses. This finding further vindicates mutational pressure as the major force driving CpG depletion among papillomaviruses. DNA methylation has been reported in several DNA viruses [35, 36] including papillomaviruses [37]. In fact, methylation of papillomavirus DNA has been shown for both integrated and episomal forms [38, 39]. While studies on polyomavirus genomes have failed to demonstrate DNA methylation [40, 41]. Our findings on the correlation between CpG dinucleotides and the gain in TpG and CpA dinucleotides among papillomaviruses or the lack of it among polyomaviruses is concurrent with reports on genomic DNA methylation among these viruses.

No major differences have been reported in the repertoire of DNA methyltransferases among vertebrates. However, among vertebrate host genomes, humans and other mammals show extensive DNA methylation (~67–80%) [42], but much lower levels of DNA methylation (<30%) are reported in birds [43, 44]. Since our results clearly demonstrate a role for DNA methylation in the evolution of papillomaviruses, we speculate that the differences in the relative abundance of CpG dinucleotides between papillomaviruses infecting humans or other mammals as compared to aves (Fig 1c) could be linked to the differences in host methylation capabilities. Other possible factors potentially contributing to the differences in CpG dinucleotide frequencies across papillomaviruses infecting different host groups include (a) Yet unknown differences in efficiencies of T/G mismatch (arising due to deamination of methylated cytosines) repair mechanisms among vertebrate hosts (b) Differences, if any, in the repertoire of cytidine deaminases such as activation-induced cytidine deaminase (AID)/apolipoprotein B RNA-editing catalytic component (APOBEC) among the different host groups.

Our findings show that polyomaviruses infecting humans or mammals are extensively CpG depleted whereas those infecting birds had modest CpG depletion (Fig 1d). We also show a major role for translational selection in the depletion of CpG dinucleotides among polyomaviruses. Possible reasons for the differences in the extent of CpG depletion among polyomaviruses infecting different host groups include (a) Differences in tRNA abundance or synonymous codon usage among the host groups (b) Differences in the duration of virus-host relationship among polyomaviruses infecting different host groups. For example, it is well known that humans and other mammals infected by polyomaviruses in general do not clear the infection; thus allowing for a long-term virus-host relationship. In contrast, polyomaviruses infecting birds usually cause acutely fatal infections that greatly reduce the duration of the virus-host relationship. Among avian polyomaviruses goose polyomaviruses are known to cause chronic infections. Interestingly, goose polyomaviruses are the most CpG depleted polyomaviruses (average CpG O/E: 0.31) among all avian polyomaviruses (average CpG O/E: 0.44); this finding supports a potential role for the duration of virus-host relationship in determining the extent of CpG depletion.

## Conclusion

Our study shows that CpG dinucleotides are the most depleted dinucleotides among papillomaviruses and polyomaviruses and CpG depletion is therefore likely to play a major role in shaping the evolution of these viruses. We also demonstrate that the extent of CpG depletion among papillomaviruses and polyomaviruses is dependent on the evolutionary lineage of the infected host. CpG dinucleotide depletion is linked to mutational pressure among papillomaviruses and to translational selection among polyomaviruses. Methylation and deamination of papillomavirus genomes may contribute at least in part to the mutational pressure leading to CpG depletion in this group of viruses. Taken together, our findings provide new perspectives on CpG dinucleotide depletion among small ds-DNA viruses infecting vertebrates and highlight the existence of fundamental differences in host-induced evolutionary pressures leading to CpG depletion.

## Supporting Information

S1 Fig GC content of papillomaviruses and polyomaviruses studied. A box plot showing the GC content of papillomaviruses and polyomaviruses. Papillomaviruses had significantly higher GC content as compared to polyomaviruses (P = 0.0047).
(TIF)

S1 Table Accession numbers of the viruses studied. (XLSX)
