# Supplementary figures and images for: Depletion of CpG Dinucleotides in Papillomaviruses and Polyomaviruses: A Role for Divergent Evolutionary Pressures
Source: PLoS One. 2015 Nov 6;10(11):e0142368. doi: 10.1371/journal.pone.0142368 (PMC4636234; doi:10.1371/journal.pone.0142368)

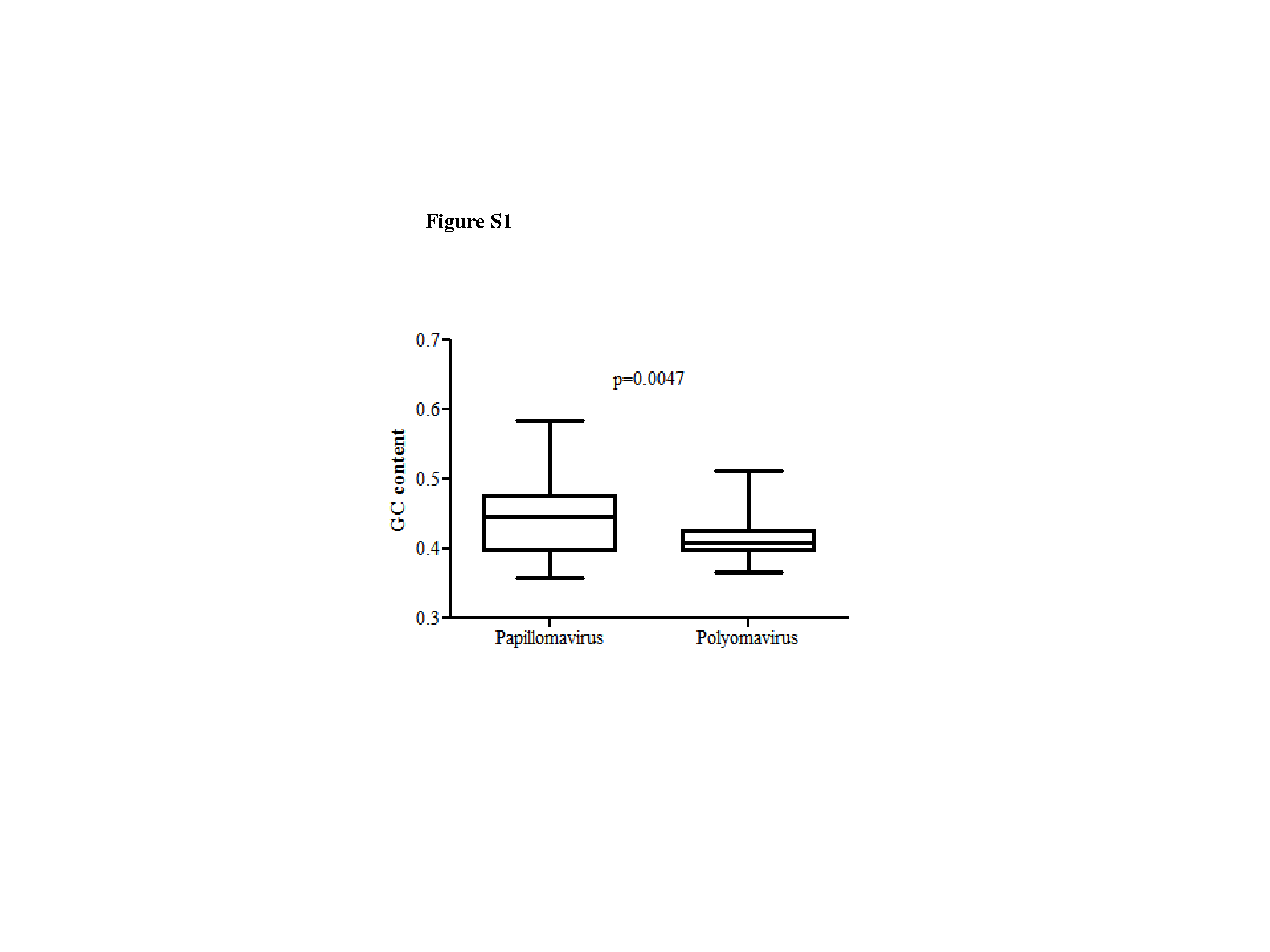

Supplement: S1 Fig — A box plot showing the GC content of papillomaviruses and polyomaviruses. Papillomaviruses had significantly higher GC content as compared to polyomaviruses (P = 0.0047). (TIF) [file pone.0142368.s001.tif]
